# Aberrant telomere length and mitochondrial DNA copy number in suicide completers

**DOI:** 10.1038/s41598-017-03599-8

**Published:** 2017-06-09

**Authors:** Ikuo Otsuka, Takeshi Izumi, Shuken Boku, Atsushi Kimura, Yuan Zhang, Kentaro Mouri, Satoshi Okazaki, Kyoichi Shiroiwa, Motonori Takahashi, Yasuhiro Ueno, Osamu Shirakawa, Ichiro Sora, Akitoyo Hishimoto

**Affiliations:** 1 Department of Psychiatry, Kobe University Graduate School of Medicine, Kobe, Japan; 2 Department of Neuropharmacology, Hokkaido University Graduate School of Medicine, Sapporo, Japan; 3 Division of Legal Medicine, Department of Community Medicine and Social Health Science, Kobe University Graduate School of Medicine, Kobe, Japan; 4 Department of Neuropsychiatry, Kindai University Faculty of Medicine, Osaka, Japan

## Abstract

Short telomere length (TL) occurs in individuals under psychological stress, and with various psychiatric diseases. Recent studies have also reported mitochondrial DNA copy number (mtDNAcn) alterations under several neuropsychiatric conditions. However, no study has examined whether aberrant TL or mtDNAcn occur in completed suicide, one of the most serious outcomes of mental illnesses. TL and mtDNAcn in post-mortem samples from 528 suicide completers without severe physical illness (508 peripheral bloods; 20 brains) and 560 samples from control subjects (peripheral bloods from 535 healthy individuals; 25 post-mortem brains) were analysed by quantitative polymerase chain reaction. Suicide completers had significantly shorter TL and higher mtDNAcn of peripheral bloods with sex/age-dependent differences (shorter TL was more remarkably in female/young suicides; higher mtDNAcn more so in male/elderly suicides). The normal age-related decline of TL and mtDNAcn were significantly altered in suicide completers. Furthermore, shorter TL and lower mtDNAcn of post-mortem prefrontal cortex were seen in suicide completers compared to controls. This study shows the first association of aberrant telomeres and mtDNA content with suicide completion. Our results indicate that further research on telomere shortening and mitochondrial dysfunction may help elucidate the molecular underpinnings of suicide-related pathophysiology.

## Introduction

Suicide is a significant public health problem that accounts for nearly 1 million deaths worldwide each year. Previous data have implicated abnormalities in the hypothalamic-pituitary-adrenal (HPA) axis, noradrenergic, and serotonergic systems in suicide^1, 2^. The involvement of genetic factors has also been demonstrated in suicidal behaviour by family studies, twin and adoption studies, candidate gene analyses and genome-wide association studies (GWAS)^3, 4^. These diverse pieces of evidence suggest that suicide is caused by the accumulation of stress, traumatic events, and/or illnesses in individuals with neurobiological changes and genetic risk. However, biological insights into suicidal behaviour lag behind those of other mental problems, and no useful genetic biomarker of suicide risk has been found. This could be because suicidal behaviour varies in terms of degree of lethality and suicidal intent, and it is very difficult to obtain viable biological samples from suicide completers, which is the most serious phenotype.

Telomeres, repetitive nucleotide sequences located at the ends of chromosomes, have gained special attention in the field of stress biomarkers. Telomere shortening to a critical length triggers genomic instability and cellular apoptosis. In somatic cells such as leukocytes, telomeres shorten with advancing age and cell divisions because of low telomerase activity^5^. Therefore, telomere length (TL) largely reflects the cellular biological age^6^. However, TL shortening has also been associated with the presence of stressors, such as various physical diseases and mental illnesses^7^. Indeed, several studies have documented short TL in individuals with psychological stress, and with various psychiatric diseases including major depressive disorder (MDD)^8, 9^, schizophrenia^10, 11^, anxiety disorders^12^, posttraumatic stress disorder (PTSD)^13^ and bipolar disorder^14^; however, these associations have not always been observed^15, 16^. Recently, a significant meta-analysis demonstrated that short TL was found in MDD, anxiety disorders and PTSD, but not in schizophrenia and bipolar disorder^17^. In addition, several reports have shown shorter telomeres in white matter oligodendrocytes, and in the hippocampus of post-mortem MDD brains^18, 19^.

Increasing interest has developed in the association of mitochondrial dysfunctions with neuropsychiatric conditions. Mitochondria are ubiquitous organelles in eukaryotic systems that play crucial roles in cellular energy production, calcium signalling, cell growth and differentiation, cell cycle control, and cell death^20^. Functional changes in mitochondria appear to be associated with aging and age-related disorders^21^. Mitochondrial biogenesis can be indirectly assessed by measuring mitochondrial DNA (mtDNA) copy number (cn), the number of mtDNA molecules per cell. Although alterations of mtDNAcn are considered an index of mitochondrial dysfunction^22^, only limited studies have examined mtDNAcn changes in psychological stress or psychiatric disorders^23–27^.

Published evidence has demonstrated the link between telomere shortening and mitochondrial dysfunction. For example, telomere shortening leads to subsequent p53-mediated repression of peroxisome proliferator-activated receptor gamma and coactivator-1α and 1β (PGC-1α and PGC-1β), which can lead to mitochondrial dysfunction^22, 28, 29^. Recently, two reports showed evidence that shorter TL and higher mtDNAcn occurred in the same cohorts with MDD or other neuropsychiatric conditions (e.g., childhood adversity)^30, 31^.

Telomere shortening and mitochondrial dysfunction have attracted much attention as key processes in cellular aging and neuropsychiatric conditions. However, no previous study has been conducted to examine whether aberrant telomeres or mtDNA are linked to completed suicide, which is the most serious outcome of psychological stress and psychiatric diseases.

Here, we report on the first investigation of TL and mtDNAcn in post-mortem bloods and brains from suicide completers without severe physical illness, and compared these results to those of control subjects.

## Results

### TL and mtDNAcn in blood samples of suicide completers and controls

**Figure 1:**
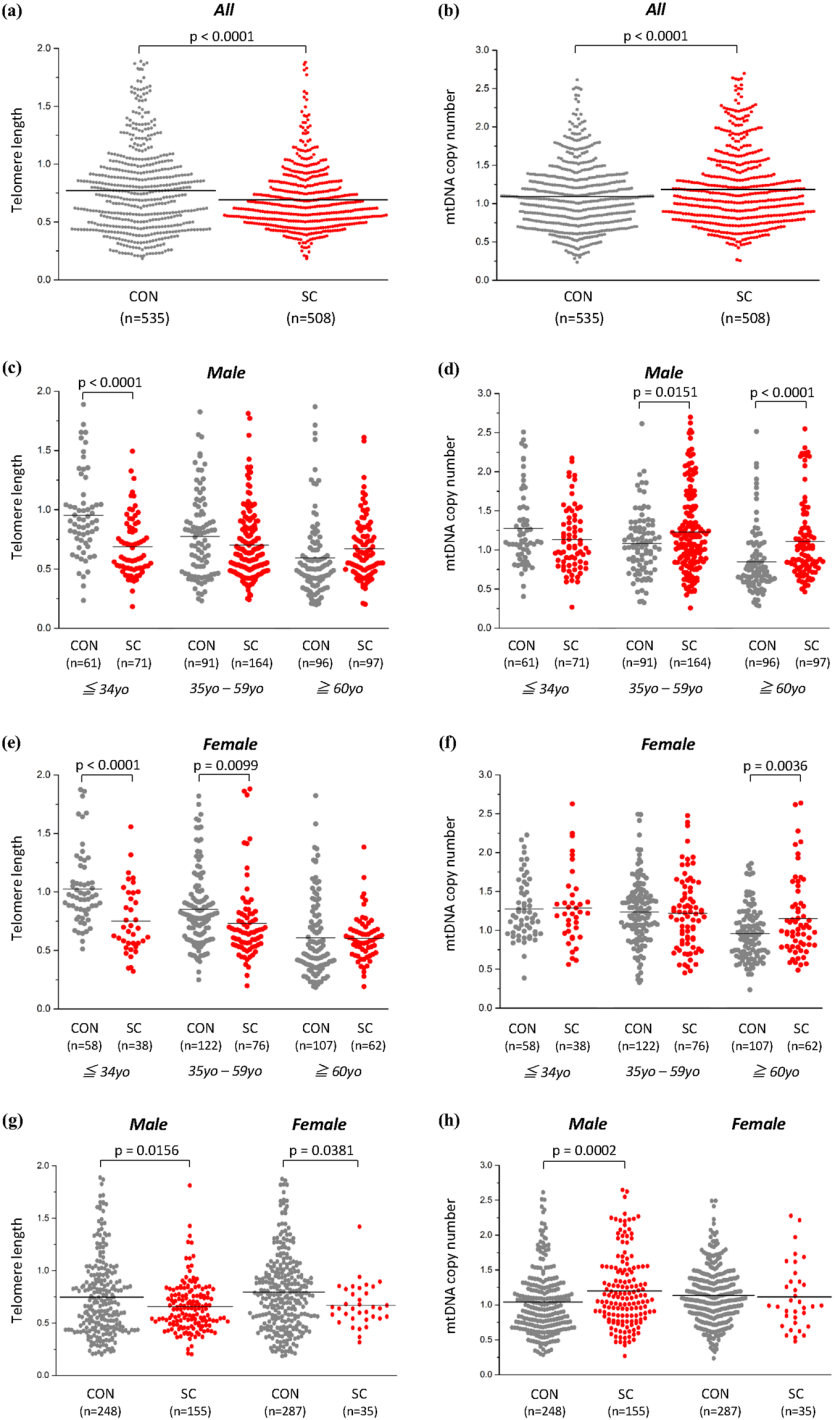
Dot plot of Telomere length (TL) and mtDNA copy number (mtDNAcn) of bloods in suicide completers and healthy controls. (**a**) TL in all suicide completers and controls. (**b**) mtDNAcn in all suicide completers and controls. (**c**) TL in male suicide completers and male controls. (**d**) mtDNAcn in male suicide completers and male controls. (**e**) TL in female suicide completers and female controls. (**f**) mtDNAcn in female suicide completers and female controls. (**g**) TL in suicide completers without psychiatric disorders and/or psychotropic medication and controls. (**h**) mtDNAcn in suicide completers without psychiatric disorders and/or psychotropic medication and controls. Figure (**c**–**f**) were divided into three age groups (≤34 years old, young; 35–59 years old, middle; ≥60 years old, elderly). All p values were adjusted by age and sex, or with age as covariates. Horizontal lines represent mean values for each group. Abbreviations: CON, control; SC, suicide; yo, years old.

The results of regression analyses of TL and mtDNAcn in blood samples of suicide completers (n = 508) and healthy controls (n = 535) are shown in Table 1. Demographic data of sex and age (≤34 years old, young; 35–59 years old, middle; ≥60 years old, elderly)-based subgroup analyses are shown in Table S1. Significantly shorter telomeres in suicide completers were observed (β = −0.0928, p < 0.0001) (Table 1 and Fig. 1a); this effect appeared to be greater in female suicide completers (male, β = −0.0684, p = 0.0618; female, β = −0.1238, p = 0.0025) (Table S1). Significantly shorter TL was seen in young suicide completers in both sexes (male, β = −0.3224, p < 0.0001; female, β = −0.3120, p < 0.0001). In middle-age suicide completers, this shortening was specific to women (male, β = 0.0786, p = 0.1546; female, β = −0.1566, p = 0.0099); it was not found at all in elderly suicide completers (Fig. 1c,e and Table S1). A significantly higher mtDNAcn than that in controls was observed in suicide completers (β = 0.1038, p < 0.0001) (Table 1 and Fig. 1b). This effect appeared to be greater in men than women (male, β = 0.1320, p = 0.0003; female, β = 0.0671, p = 0.0584) (Table S1). Elderly suicide completers had significantly higher mtDNAcn than the other age groups in both sexes (male, β = 0.2750, p < 0.0001; female, β = 0.1855, p = 0.0036), whereas this increase was specific to middle-aged men (male, β = 0.1309, p = 0.0151; female, β = −0.0114, p = 0.8282) and absent in young suicide completers (Fig. 1d,f and Table S1). Even when suicide completers with current/past psychiatric disorders and/or psychotropic medication use were excluded, TL was significantly shorter for the suicide completers (male, β = −0.1081, p = 0.0156; female, β = −0.1595, p = 0.0381) than the controls, and male, but not female, suicide completers showed a significantly higher mtDNAcn (male, β = 0.1639, p = 0.0002; female, β = −0.0123, p = 0.8463) (Fig. 1g,h; The results of regression analyses are shown in Table S2). We gathered the presence/absence of suicide attempt history for 471 suicide completers, and conducted an additional analysis among only those samples. However, there was no association between suicide attempt history and either TL or mtDNAcn (Table S3). Furthermore, we investigated whether or not TL and mtDNAcn were biochemically stable post-mortem. There was no association between post-mortem interval (PMI) and TL or mtDNAcn in post-mortem blood samples (Table S4).

**Table 1:** Regression analyses of telomere length and mtDNA copy number of blood in suicide completers and controls.

	Telomere length				mtDNA copy number			
	β^a^	s.e.	t	p valueb	β^a^	s.e.	t	p value^b^
***Total samples (n*** = ***1043); Suicide completers (n*** = ***508) and Controls (n*** = ***535)***								
—Phenotype (Suicide vs. Control)	−0.0928	0.0272	−3.411	**<0.0001 **	0.1038	0.0255	4.078	**<0.0001**
—Age	−0.0077	0.0008	−9.950	**<0.0001**	−0.0048	0.0007	−6.631	**<0.0001**
—Sex (Male vs. Female)	−0.0358	0.0274	−1.308	0.1912	−0.0703	0.0256	−2.745	**0.0062**

Abbreviation: s.e., standard error. ^a^β means regression coefficient derived from generalized linear models. ^b^p values shown in bold are significant at <0.05.

There was a weak trend of sex effect on TL (p = 0.0663), and a statistically significant effect on mtDNAcn (p = 0.0016) in healthy controls; no sex effect on either TL or mtDNAcn was found in suicide completers (Table S5).

**Figure 2:**
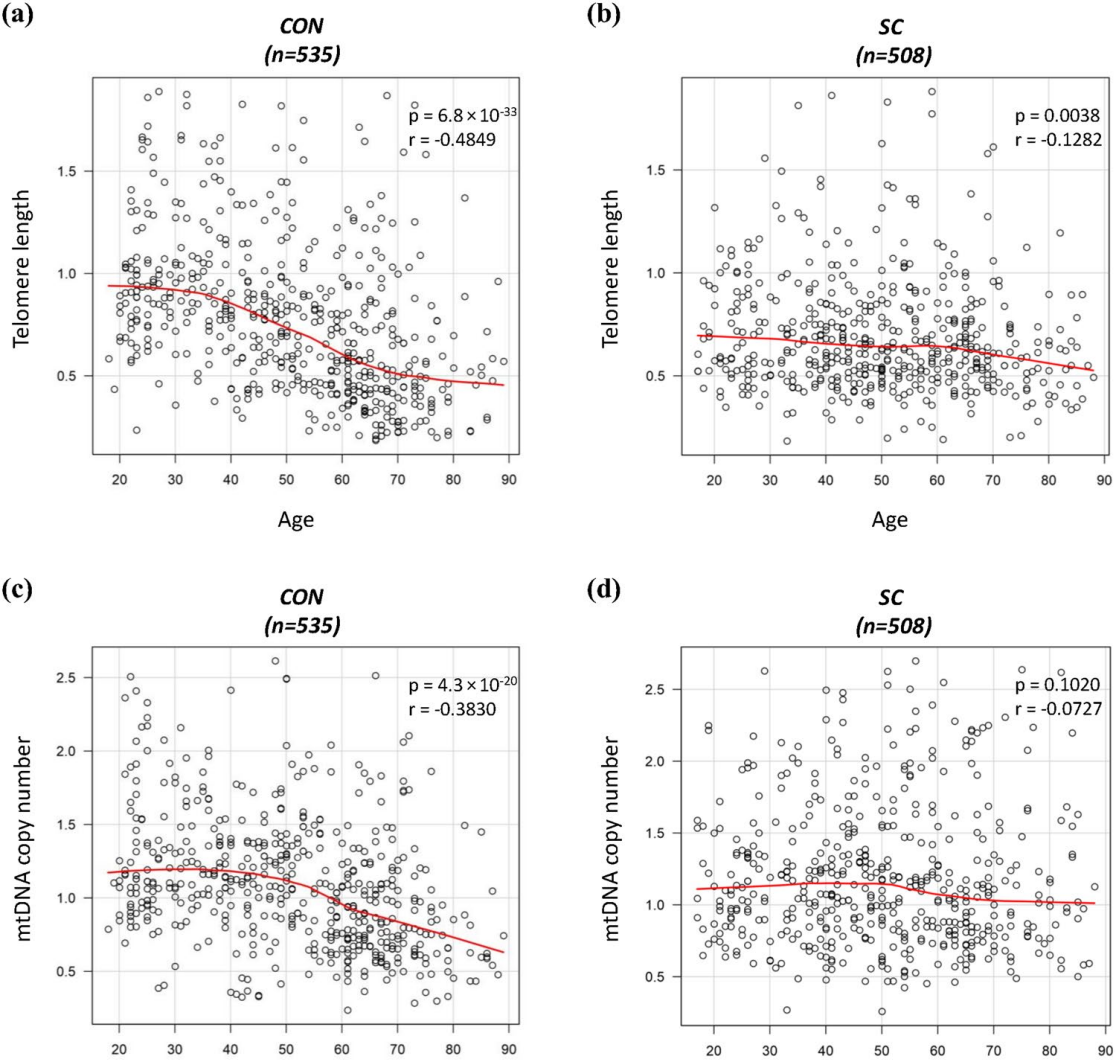
Age-TL/mtDNAcn relationship in suicide completers and healthy controls. (**a**) Correlation between TL and age in controls. (**b**) Correlation between TL and age in suicide completers. (**c**) Correlation between mtDNAcn and age in controls. (**d**) Correlation between mtDNAcn and age in suicide completers. Red line fitting a smooth spline showed non-linear relationship between TL/mtDNAcn and age. All p values and r values were calculated by Spearman’s rho tests. Abbreviations: CON, control; SC, suicide.

Healthy controls showed the normal age-related reduction in TL and mtDNAcn (TL, r = −0.4849, p < 0.0001; mtDNAcn, r = −0.3830, p < 0.0001) (Fig. 2a,c). In suicide completers, however, age-related attrition was significantly milder compared to controls (r = −0.1282, p = 0.0038), and the negative correlation between mtDNAcn and age was not observed (r = −0.0727, p = 0.1020) (Fig. 2b,d).

### TL and mtDNAcn in brain samples of suicide completers and controls

**Figure 3:**
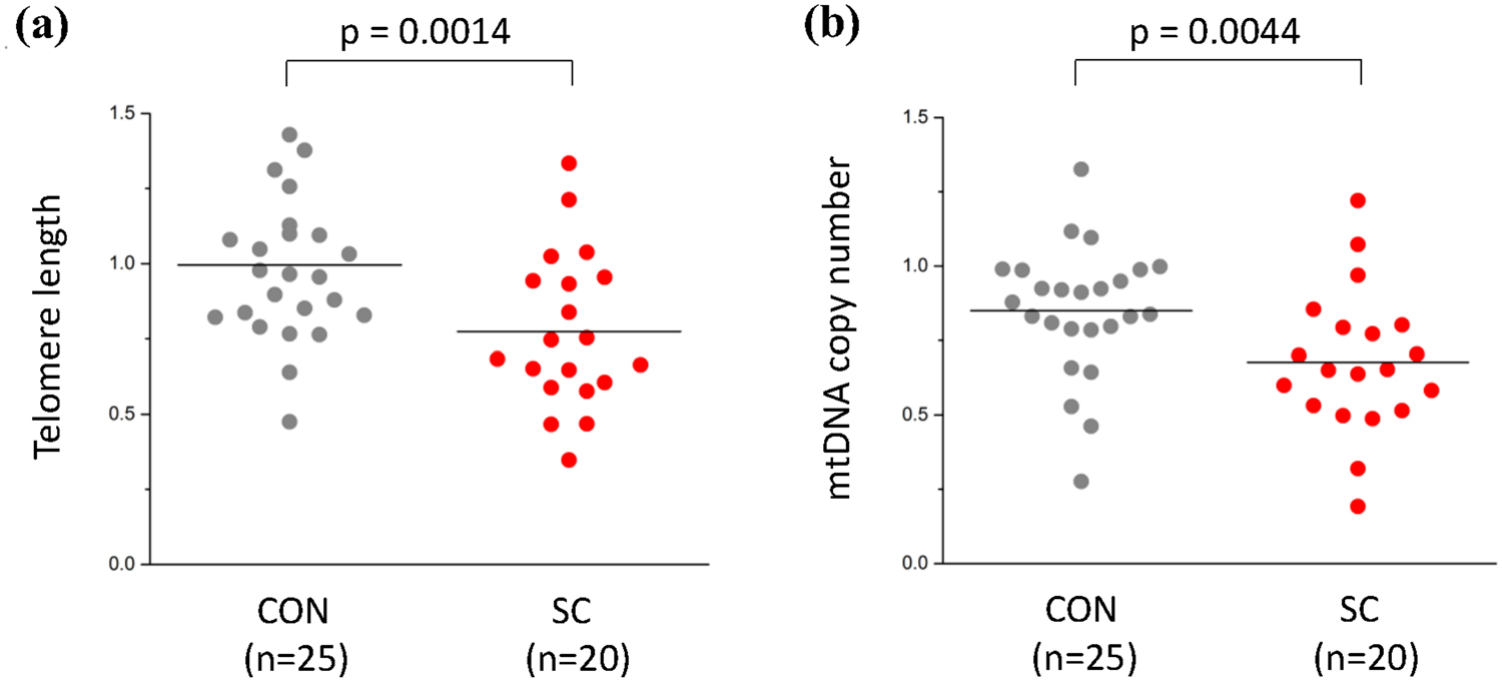
Dot plot of Telomere length (TL) and mtDNA copy number (mtDNAcn) of post-mortem dorsolateral prefrontal cortex (DLPFC) in suicide completers and controls. (**a**) TL of DLPFC in suicide completers and controls. (**b**) mtDNAcn of DLPFC in suicide completers and controls. All p values were adjusted by age, sex and post-mortem interval as covariates. Horizontal lines represent mean values for each group. Abbreviations: CON, control; SC, suicide.

Regression analyses of TL and mtDNAcn in dorsolateral prefrontal cortex (DLPFC) samples of suicide completers (n = 20) and controls (n = 25) are shown in Table 2. We noted a significantly shorter TL and lower mtDNAcn in the DLPFC of suicide completers compared to controls (β = −0.3006, p = 0.0014 and β = −0.2803, p = 0.0044, respectively) (Fig. 3a,b). Regression analysis showed that PMI had no significant effect on TL and mtDNAcn in any brain sample (Table 2).

**Table 2:** Regression analyses of telomere length and mtDNA copy number of post-mortem dorsolateral prefrontal cortex (DLPFC) in suicide completers and controls.

	Telomere length				mtDNA copy number			
	β^a^	s.e.	t	p value^b^	β^a^	s.e.	t	p value^b^
***Total samples (n = 45); Suicide completers (n = 20) and Controls (n = 25)***								
—Phenotype (Suicide vs. Control)	−0.3006	0.0875	−3.436	**0.0014**	−0.2803	0.0929	−3.018	**0.0044**
—Age	−0.0064	0.0026	−2.462	**0.0182**	−0.0045	0.0028	−1.633	0.1102
—Sex (Male vs. Female)	−0.0552	0.0918	−0.601	0.5510	−0.1333	0.0975	−1.368	0.1790
—PMI	0.0035	0.0050	0.707	0.4835	−0.0001	0.0053	−0.006	0.9951

Abbreviations: PMI, post-mortem interval; s.e., standard error. ^a^β means regression coefficient derived from generalized linear models. ^b^p values shown in bold are significant at <0.05.

## Discussion

This is the first study to investigate TL and mtDNAcn in post-mortem blood and brain samples of suicide completers. First, we found significantly shorter TL and higher mtDNAcn in blood samples from suicide completers, which remained significant after excluding subjects with psychiatric disorders and/or psychotropic medication use. Our results were similar to prior findings of shortened TL and increased mtDNAcn in cohorts with MDD or other neuropsychiatric conditions^30, 31^. The results from one of these studies revealed two additional findings in laboratory mice: (i) behavioural stress led to decreased TL and increased mtDNAcn, and (ii) mice injected with corticosterone showed shortened telomeres and increased mtDNAcn following behavioural stress^30^. According to other findings, high cortisol reactivity to stress is associated with shorter telomeres in children of depressed mothers^32^. Cortisol also inhibits telomerase activity *in vitro* ^33^. These data suggest that alterations of TL and mtDNA content after stress are at least partially caused by activation of the HPA axis. Several lines of evidence, especially resistance to the dexamethasone suppression test (DST), also indicate that HPA axis abnormalities might be the main diathesis for suicide^2^. Further investigation of the HPA axis may help clarify the association between alterations of TL/mtDNAcn and suicide. In fact, a recent report showed significantly higher plasma levels of free-circulating mtDNA in medication-free suicide attempters compared to healthy controls; notably, the higher free plasma mtDNA levels were associated with resistance to the DST in suicide attempters^34^.

In addition, we found significant sex-dependent differences in aberrant TL/mtDNAcn. Shorter TL was more divergent from controls in female suicide completers, and the significantly higher mtDNAcn was higher still in male suicides. These sex-dependent differences likely led to the loss of the sex effects on blood cell TL and mtDNAcn in suicide completers. Similarly to our control samples, previous studies in the general population reported longer TL in women than in men, and a slower rate of telomere attrition in women^35, 36^. This difference is thought to be partially related to female hormones such as estrogen, which may reduce oxidative stress and stimulate the production of telomerase^37, 38^. Other reports showed the relationship between lower levels of progesterone and susceptibility to suicide attempts in women^39, 40^. Further research into telomere shortening and disturbed levels of female hormones in suicide will be needed to better elucidate the underlying mechanism of this association. In studying mtDNA, several studies of the general population showed a higher level of mtDNA content in women than in men, consistent with our findings in healthy controls^41, 42^. In addition, Borras *et al*. reported that female rats were less prone to mtDNA damage by reactive oxygen species^43^. These characteristics of female mtDNA may lead to the less significant results found in this study.

We also found age-dependent differences in aberrant TL/mtDNAcn; shorter TL was significant only in suicide completers under 60 years old, particularly in the young (under 34 years old), and higher mtDNAcn was far more remarkable in elderly suicide completers. These results indicated that the biological basis for completed suicide appears to be different between young, middle-aged, and elderly individuals. In addition, aberrant TL/mtDNAcn in completed suicide may be more easily detectable in comparison with healthy young people with longer telomeres and healthy elderly with lower mtDNA content, respectively.

We also performed further investigation about the association of age with TL and mtDNAcn. The results of many studies have shown an age-related decline in TL^29, 44^; several studies have shown that mtDNAcn did not decline until around 50 years of age, after which an age-related decline was observed in healthy subjects^42, 45^. In our study, the control subjects also showed an age dependency for both TL and mtDNAcn similar to that seen in previous reports. In suicide completers, however, TL was significantly less correlated with age compared to healthy controls, and mtDNAcn showed no negative association. These altered correlations were at least partially caused by the presence of young suicide completers with shorter TL, and elderly suicide completers with higher mtDNAcn.

DLPFC is one of the brain regions which previous studies indicate is related to suicide vulnerability. Structural and functional neuroimaging studies in suicidal populations revealed abnormal volume reductions and dysfunction in the frontal cortex, primarily in the DLPFC as well as the orbitofrontal cortex (OFC) and ventrolateral PFC^46^. The DLPFC plays an important role in cognitive control and emotional regulation, both of which are involved in suicide-related pathophysiology^47^. In addition, elevated corticotropin-releasing hormone levels have been found in the post-mortem DLPFC of patients who committed suicide relative to controls, which suggests that HPA axis hyperactivity was present^48^. In this study, we identified shorter telomeres in the post-mortem DLPFC of suicide completers. A previous study reported that TL was not changed in the post-mortem DLPFC region of patients with MDD, contrary to our findings^19^. These inconsistent results may derive in part from the difference between MDD and completed suicide, in that the severity of or duration of exposure to the psychological stressor causing suicidal ideation may have been greater in patients who completed suicide, which thus increased the shortening effect. Interestingly, one large cohort study showed that peripheral blood cell TL, but not brain tissue TL, was positively associated with total cerebral volume, as well as that of some distinct regions in the PFC^49^. In future studies, it would be interesting to analyse this direct association between brain region/cell-specific telomere length and brain volume in the post-mortem brains of both of suicide completers and controls. We also found lower mtDNA content in the post-mortem DLPFC of suicide completers. Hunter *et al*. reported that acute stress in adult rats induced down-regulation of mtDNA-encoded genes, and that corticosteroids regulated rat hippocampal mtDNA gene expression via glucocorticoid receptors^50^. They also determined that the glucocorticoid receptor can translocate into mitochondria in neurons of the PFC^51^. HPA axis abnormalities in the brains of suicidal individuals may cause the lower mtDNA content of the PFC reported here. Only a few studies have investigated mtDNA content in human brain samples from patients with psychiatric disorders (MDD, schizophrenia and bipolar disorder), and no differences were reported relative to control samples in any of these studies^52–54^. In addition, no study provides clues as to why the data herein demonstrated disturbances in mtDNAcn that were the opposite of those in blood and DLPFC in this study. Further studies focused on mtDNA content and psychiatric conditions in distinct brain regions are required to clarify these findings.

A major strength of this study is the large sample size of suicide completers. Indeed, most previous candidate gene analyses and GWAS for completed suicide were limited by a small sample size^2, 4, 55^. In addition, we obtained information regarding age, comorbid diseases (severe physical illness and psychiatric disease) and psychotropic medication use, to exclude factors that can alter TL and mtDNAcn as much as possible. Therefore, the significant alterations in TL and mtDNAcn reported here should be robustly linked to the molecular signature of suicidal behaviour with the intention of completion. However, there are some limitations to consider as well. First, the condition of post-mortem samples should be considered. Although we selected only subjects with certain clinical information prior to death, the influence of PMI, pH changes and protein degradation on our results cannot be controlled. In this study, there was no association between PMI and TL or mtDNAcn in post-mortem blood and brain samples. Furthermore, both the shorter telomeres and aberrant mtDNAcn in suicide completers reported here showed significant sex-dependent differences. According to one forensic study, the age-related decline of TL also remained strong in healthy cadaver blood^56^. These findings may provide evidence that our results were not influenced by the acquisition of samples post-mortem. Second, the criteria underlying the exclusion of psychiatric cases from analysis are insufficient, since we only investigated the cases through their medical records and bereaved family interviews. Thus, some suicide completers who we considered to be without psychiatric cases might have indeed experienced psychiatric symptoms that met the criteria of specific disorders. Third, we could not exclude other potential confounders (e.g. smoking status, BMI) that are known to affect TL and mtDNAcn as well^57–59^. Fourth, TL and mtDNAcn of other brain regions correlated with suicidal vulnerability (e.g., OFC, ventral PFC, hippocampus or amygdala) should be explored in the post-mortem brains of suicide completers.

In conclusion, we report the first association of aberrant telomeres and mtDNAcn with suicide completion. Our results raise the possibility that further research into telomere shortening and mtDNA dysfunction may elucidate the molecular underpinnings of suicide-related pathophysiology.

## Methods

### Subjects

The entire study design and procedures were performed in accordance with the Declaration of Helsinki. This study was approved by the Ethical Committee for Genetic Studies of the Kobe University Graduate School of Medicine. All subjects were of Japanese descent and ranged from 18 years to 89 years of age. Autopsies on suicide victims were conducted at the Division of Legal Medicine in the Department of Community Medicine and Social Health Science at the Kobe University Graduate School of Medicine. The verdict of “completed suicide” was made through discussion with the Medical Examiner’s Office of Hyogo Prefecture and the Division of Legal Medicine in the Kobe University Graduate School of Medicine. Informed consent was obtained from all of the participants and from the families of the subjects used for post-mortem blood and brain experiments.

### Blood and brain sampling

Peripheral blood samples were obtained from all of suicide completers post-mortem (n = 508), and from healthy living controls (n = 535). The demographics of the subjects are shown in Table S6. The age distribution was matched among suicide completers and controls in each sex group. We excluded suicide victims with severe physical illnesses (cardiovascular diseases, cerebral infarction, diabetes, bone marrow diseases, and cancer) because they can affect TL and mtDNAcn^60–65^. All of the healthy volunteers were recruited from the main islands of Japan, including medical students, hospital workers, and the general population. No control subjects were related to each other, or manifested psychiatric problems in unstructured interviews conducted by two psychiatrists using the Diagnostic and Statistical Manual of Mental Disorders 4th edition (DSM-IV) criteria. During the interview, all control subjects were checked for a personal and family history of psychiatric disorders and/or suicidal behaviours. We excluded control subjects with personal and/or familial history of psychiatric disorders and/or suicidal behaviours.

Autopsied brains were obtained from 20 suicide victims and 25 control subjects. Demographic data are shown in Table S7. DLPFC was dissected on dry ice for subsequent DNA extraction.

### Measurement of TL and mtDNAcn

Blood and brain samples were stored at −80 °C before use. DNA was extracted using the QIAamp DNA Blood Midi Kit and DNeasy Blood & Tissue Kit (Qiagen Inc., Valencia, CA) as appropriate. Each DNA sample was quantified and qualified using a NanoDrop spectrophotometer (Thermo Scientific, Wilmington, DE).

TL was measured using quantitative polymerase chain reaction (qPCR), by applying the telomere/single-copy gene ratio method^66^ with minor modifications. Briefly, the method was used to measure the ratio between the number of telomere repeats and that of a single-copy gene (β-haemoglobin [HGB]) used as a quantitative control, relative to a reference sample. mtDNAcn was calculated based on measuring the amount of mtDNA (NADH dehydrogenase, subunit 1 [ND1]) relative to that of a nuclear gene (HGB). All qPCR experiments were performed using a 7500 Real-Time PCR System (Applied Biosystems, Foster City, CA), with SYBR Green Master Mix (Applied Biosystems, Foster City, CA). The primer sequences and cycling conditions are described in Table S8. Each sample was run in triplicate, using 10 ng of DNA. Amplification of telomeres, ND1, and the single-copy gene HGB were performed in separate runs, using the same reference sample in the same well positions. TL and mtDNAcn were determined by measuring the ratio of telomeric and mtDNA content to that of a reference single-copy gene in each sample, relative to the reference gene; the standard curve method using a 5-point serial-dilution series with reference DNA was employed. Laboratory personnel were blinded with regard to case-control status and the sample order was randomized in each batch.

### Statistical analysis

Statistical analysis was performed using R Version 3.2.2 and EZR^67, 68^. Student’s *t*-tests were performed to analyse between-group comparisons of the continuous variables. Regression analyses using generalized linear models with a gamma distribution and log link were applied to analyse between-group comparisons of TL and mtDNAcn, with covariates (age, sex, suicide attempt history and PMI) as needed. Spearman’s rho tests were performed to assess the relationships between TL/mtDNAcn and age. Dummy variables were used as necessary (phenotype, control = 0 and suicide = 1; sex, male = 0 and female = 1; suicide attempt history, no = 0 and yes = 1). Statistical significance was defined as two-tailed p < 0.05.

### Data availability

All data generated or analysed during this study are included in this published article (and its Supplementary Information files).

## Electronic supplementary material

Supplementary information
