# Supplementary material for: Aberrant telomere length and mitochondrial DNA copy number in suicide completers
Source: Sci Rep. 2017 Jun 9;7:3176. doi: 10.1038/s41598-017-03599-8 (PMC5466636; doi:10.1038/s41598-017-03599-8)
Supplement: Supplementary file 1 — Supplementary information [file 41598_2017_3599_MOESM1_ESM.pdf]

Supplementary Information

Aberrant telomere length and mitochondrial DNA copy number in suicide completers

Ikuo Otsuka, M.D., Ph.D.; Takeshi Izumi, M.D., Ph.D.; Shuken Boku, M.D., Ph.D.; Atsushi Kimura, M.D.; Yuan Zhang, M.D.; Kentaro Mouri, M.D., Ph.D.; Satoshi Okazaki, M.D., Ph.D.; Kyoichi Shiroiwa, M.D., Ph.D.; Motonori Takahashi, M.D., Ph.D.; Yasuhiro Ueno, M.D., Ph.D.; Osamu Shirakawa, M.D., Ph.D.; Ichiro Sora, M.D., Ph.D.; Akitoyo Hishimoto*, M.D., Ph.D.

Table S1.

Subgroup analyses of telomere length and mtDNA copy number of blood in suicide completers and controls.

	Telomere length				mtDNA copy number			
	β[a]	s.e.	t	p value[b]	β[a]	s.e.	t	p value[b]
Different sex group (n: Suicide and Control)								
Male (332 and 248)								
- Phenotype (Suicide vs. Control)	-0.0684	0.0366	-1.871	0.0618	0.1320	0.0360	3.663	**0.0003**
- Age	-0.0063	0.0010	-6.058	**<.0001**	-0.0049	0.0010	-4.735	**<.0001**
Female (176 and 287)								
- Phenotype (Suicide vs. Control)	-0.1238	0.0407	-3.041	**0.0025**	0.0671	0.0353	1.897	0.0584
- Age	-0.0093	0.0011	-8.186	**<.0001**	-0.0047	0.0010	-4.721	**<.0001**
Different sex/age group								
Age (n: Suicide and Control)								
≤ 34								
Male (71 and 61)								
- Phenotype (Suicide vs. Control)	-0.3224	0.0647	-4.981	**<.0001**	-0.1074	0.0649	-1.655	0.1003
- Age	0.0038	0.0069	0.545	0.5870	0.0025	0.0070	0.351	0.7264
Female (38 and 58)								
- Phenotype (Suicide vs. Control)	-0.3120	0.0712	-4.379	**<.0001**	0.0109	0.0704	0.155	0.8770
- Age	0.0006	0.0078	0.079	0.9371	0.0001	0.0077	0.010	0.9925

	Telomere length				mtDNA copy number			
	β[a]	s.e.	t	p value[b]	β[a]	s.e.	t	p value[b]
35 - 59								
Male (164 and 91)								
- Phenotype (Suicide vs. Control)	-0.0786	0.0551	-1.428	0.1546	0.1309	0.0535	2.447	**0.0151**
- Age	-0.0132	0.0038	-3.484	**0.0006**	-0.0010	0.0037	-0.272	0.7855
Female (76 and 122)								
- Phenotype (Suicide vs. Control)	-0.1566	0.0601	-2.607	**0.0099**	-0.0114	0.0526	-0.218	0.8282
- Age	-0.0062	0.0042	-1.472	0.1427	-0.0018	0.0037	-0.490	0.6250
≥ *60*								
Male (97 and 96)								
- Phenotype (Suicide vs. Control)	0.1108	0.0692	1.600	0.1110	0.2750	0.0679	4.049	**<.0001**
- Age	-0.0082	0.0050	-1.649	0.1007	-0.0083	0.0049	-1.710	0.0888
Female (62 and 107)								
- Phenotype (Suicide vs. Control)	0.0250	0.0797	0.314	0.7547	0.1855	0.0628	2.952	**0.0036**
- Age	-0.0085	0.0053	-1.611	0.1090	-0.0005	0.0042	-0.115	0.9088

Abbreviation: s.e., standard error.

[a]β means regression coefficient derived from generalized linear models.

[b]p values shown in bold are significant at < 0.05.

Table S2.

Telomere length and mtDNA copy number of blood in suicide completers without psychiatric disorders and/or psychotropic medication and controls.

	Telomere length				mtDNA copy number			
	β[a]	s.e.	t	p value[b]	β[a]	s.e.	t	p value[b]
Suicides without psychiatric disorders and/or psychotropic medication and controls								
(n: Suicide and Control)								
All (190 and 535)								
- Phenotype (Suicide vs. Control)	-0.1202	0.0383	-3.138	**0.0018**	0.1218	0.0355	3.431	**0.0006**
- Age	-0.0099	0.0009	-10.77	**<.0001**	-0.0066	0.0009	-7.778	**<.0001**
- Sex (Male vs. Female)	-0.0589	0.0339	-1.736	0.0830	-0.0712	0.0314	-2.268	**0.0236**
Male (155 and 248)								
- Phenotype (Suicide vs. Control)	-0.1081	0.0446	-2.427	**0.0156**	0.1639	0.0443	3.701	**0.0002**
- Age	-0.0088	0.0012	-7.148	**<.0001**	-0.0070	0.0012	-5.691	**<.0001**
Female (35 and 287)								
- Phenotype (Suicide vs. Control)	-0.1595	0.0766	-2.082	**0.0381**	-0.0123	0.0632	-0.195	0.8463
- Age	-0.0113	0.0014	-8.152	**<.0001**	-0.0062	0.0011	-5.429	**<.0001**

Abbreviation: s.e., standard error.

[a]β means regression coefficient derived from generalized linear models.

[b]p values shown in bold are significant at < 0.05.

Table S3. Association between telomere length/mtDNA copy number and suicide attempt history.

	Telomere length				mtDNA copy number			
	β[a]	s.e.	t	p value[b]	β[a]	s.e.	t	p value[b]
Total samples (n = 471); SA (n = 97) and non-SA (n = 374)								
- Phenotype (SA vs. non-SA)	-0.0195	0.0458	-0.425	0.6708	-0.0075	0.0486	-0.154	0.8784
- Age	-0.0031	0.0010	-2.976	**0.0031**	-0.0014	0.0011	-1.254	0.2110
- Sex (Male vs. Female)	-0.0172	0.0387	-0.445	0.6567	-0.0489	0.0411	-1.189	0.2349

Abbreviations: s.e., standard error; SA, suicides with suicide attempt history; non-SA, suicides without suicide attempt history.

[a]β means regression coefficient derived from generalized linear models.

[b]p value shown in bold is significant at < 0.05.

Table S4. Association between telomere length/mtDNA copy number and post-mortem interval in the post-mortem blood samples.

	Telomere length				mtDNA copy number			
	β[a]	s.e.	t	p value[b]	β[a]	s.e.	t	p value[b]
Suicide completers in which PMI data were available (n = 483)								
- PMI	0.0011	0.0011	1.013	0.3114	-0.0003	0.0011	-0.273	0.7852
- Age	-0.0031	0.0010	-2.987	**0.0030**	-0.0010	0.0011	-0.916	0.3599
- Sex (Male vs. Female)	-0.0136	0.0373	-0.366	0.7149	-0.0492	0.0394	-1.250	0.2120

Abbreviations: PMI, post-mortem interval; s.e., standard error.

[a]β means regression coefficient derived from generalized linear models.

[b]p value shown in bold is significant at < 0.05.

Table S5. Analyses of sex effect on telomere length/mtDNA copy number in suicide completers and controls.

	Telomere length				mtDNA copy number			
	β[a]	s.e.	t	p value[b]	β[a]	s.e.	t	p value[b]
Control (n = 535)								
- Sex (Male vs. Female)	-0.0727	0.0395	-1.840	0.0663	-0.1048	0.0331	-3.166	**0.0016**
- Age	-0.0119	0.0011	-10.59	**<.0001**	-0.0085	0.0009	-8.977	**<.0001**
Suicide completers (n = 508)								
- Sex (Male vs. Female)	-0.0008	0.0371	-0.023	0.9818	-0.0320	0.0386	-0.829	0.4073
- Age	-0.0029	0.0010	-2.826	**0.0049**	-0.0008	0.0011	-0.764	0.4450

Abbreviation: s.e., standard error.

[a]β means regression coefficient derived from generalized linear models.

[b]p values shown in bold are significant at < 0.05.

Table S6. Demographics of the subjects in peripheral blood study.

		Suicide completers (n = 508)	Healthy controls (n = 535)	p[a]
All	Average age (±s.d.)	49.9 (17.2)	50.8 (17.5)	0.383
	PMI (±s.d.)[b]	20.3 (10.5)		
Male	Number of samples	n = 332	n = 248	0.452
	Average age (±s.d.)	49.5 (16.6)	50.6 (18.2)	
	Suicide method			
	Neck hanging	233		
	Jumping from heights	48		
	Drowning	4		
	Overdosing	5		
	Self-inflicted penetrating wounds	3		
	Gas suffocation	21		
	Jumping in front of vehicles	4		
	Self-burning	2		
	Others	12		
	Comorbid psychiatric disease			
	Mood disorders	82		
	Psychotic disorders	22		
	Anxiety disorders	9		
	Personality disorders	2		
	Alcohol/Substance use disorders	6		
	Others	2		
	Unknown	54		
	Psychotropic medication use	114		
	Suicide attempt history (yes, Y; no, N; unknown, U)	Y:40, N:267, U:25		
Female	Number of samples	n = 176	n = 287	0.796
	Average age (±s.d.)	50.6 (18.3)	51.0 (16.8)	
	Suicide method			
	Neck hanging	113		
	Jumping from heights	35		
	Drowning	3		
	Overdosing	2		
	Self-inflicted penetrating wounds	3		
	Gas suffocation	8		
	Jumping in front of vehicles	1		
	Self-burning	0		
	Others	11		
	Comorbid psychiatric disease			
	Mood disorders	95		
	Psychotic disorders	11		
	Anxiety disorders	13		
	Personality disorders	5		
	Alcohol/Substance use disorders	2		
	Others	8		
	Unknown	7		
	Psychotropic medication use	117		
	Suicide attempt history (yes, Y; no, N; unknown, U)	Y:57, N:107, U:12		

Abbreviations: s.d., standard deviation; PMI, post-mortem interval.

[a] p values were calculated using student's *t*-test.

[b] PMI data were only available in 483 suicide completers.

Table S7. Demographics of the subjects in post-mortem brain study.

Phenotype	Sex (n)	Average age (±s.d.)	Comorbid psychiatric disease	PMI (±s.d.)	pH (±s.d.)
Control	Male (n=19)<br>Female (n=6)	57.9 (± 16.9)		16.5 ± 9.4	6.24 ± 0.34
Suicide	Male (n = 11)<br>Female (n = 9)	52.4 (± 14.8)	Mood disorders (n = 6)<br>Psychotic disorders (n = 3)<br>Anxiety disorders (n = 1)<br>Unknown (n= 2)	16.9 ± 7.1	6.50 ± 0.20

Abbreviations: s.d., standard deviation; PMI, post-mortem interval.

Table S8. Primer sequences and polymerase chain reaction conditions.

Locus	Primers (5'-3')
Telomere	F: GGTTTTTGAGGGTGAGGGTGAGGGTGAGGGTGAGGGT<br>R: TCCCGACTATCCCTATCCCTATCCCTATCCCTATCCCTA<br>25 cycles of 95°C for 15 s, 56°C for 1 min
Single copy gene (HGB)	F: GCTTCTGACACAACTGTGTTCACTAGC<br>R: CACCAACTTCATCCACGTTCACC<br>40 cycles of 95°C for 15 s, 58°C for 20 s, 72°C for 20 s
mtDNA (ND1)	F: AACATACCCATGGCCAACCT<br>R: AGCGAAGGGTTGTAGTAGCCC<br>40 cycles of 95°C for 15 s, 58°C for 20 s, 72°C for 20 s

Abbreviations: HGB, β-haemoglobin; min, minutes; ND1, NADH dehydrogenase subunit 1; s, seconds.
